# Takotsubo cardiomyopathy and pituitary apoplexy: a case report

**DOI:** 10.1186/s12872-020-01521-1

**Published:** 2020-05-19

**Authors:** Chun Yang, Xiu Han, Yuan Du, Ai-qun Ma

**Affiliations:** Department of Cardiovascular Medicine, First Affiliated Hospital of Xi’an Jiaotong University, Xi’an, 710061 Shaanxi China

**Keywords:** Takotsubo cardiomyopathy, Takotsubo, Stress cardiomyopathy, Pituitary apoplexy, Hypopituitarism

## Abstract

**Background:**

Takotsubo cardiomyopathy (TTC) has been widely recognized in recent decades and is triggered by either physical or psychological stressors.

**Case presentation:**

A 70-year-old woman presented to the Emergency Department due to confusion, hypotension, fever, chills, and cough. She had a one-year history of diabetes insipidus. Pituitary function examination at admission revealed decreased thyroid, sex and adrenal hormones. Pituitary MRI displayed findings suggestive of nonhemorrhagic pituitary apoplexy. Electrocardiogram (ECG) revealed T-wave inversion and extended QT interval. Transthoracic echocardiogram (TTE) showed left ventricular apical dysplasia and ballooning, accompanied by reduced left ventricular ejection fraction. Coronary angiography (CAG) revealed no obvious coronary arterial stenosis. The left ventriculogram demonstrated an octopus clathrate appearance. Most ECG and TTE changes recovered 10 days later.

**Conclusions:**

To the best of our knowledge, this is the first report of newly diagnosed TTC associated with pituitary apoplexy.

## Background

Takotsubo cardiomyopathy (TTC) was first described in Japanese in 1990 by Sato [1] because of the resemblance between Takotsubo (name of a Japanese octopus trap) and the left ventricular appearance during systole of the patients [2]. After the first report, the syndrome has been widely recognized. In 2001, more cases were described in English by Tsuchihashi K [3]. Since then, the disease has acquired multiple names, such as stress cardiomyopathy, apical ballooning syndrome and broken heart syndrome [4]. TTC is triggered by physical or emotional stressors and is characterized by a reduction in the left ventricular ejection fraction (LVEF) associated with a balloon-like wall motion abnormality, typical hypokinesia in the apical segment and hyperkinesia in the basal segments in the absence of significant coronary artery diseases [5–7]. TTC is a transient LV dysfunction and generally recovers within a few days to weeks.

## Case presentation

**Fig. 1:**
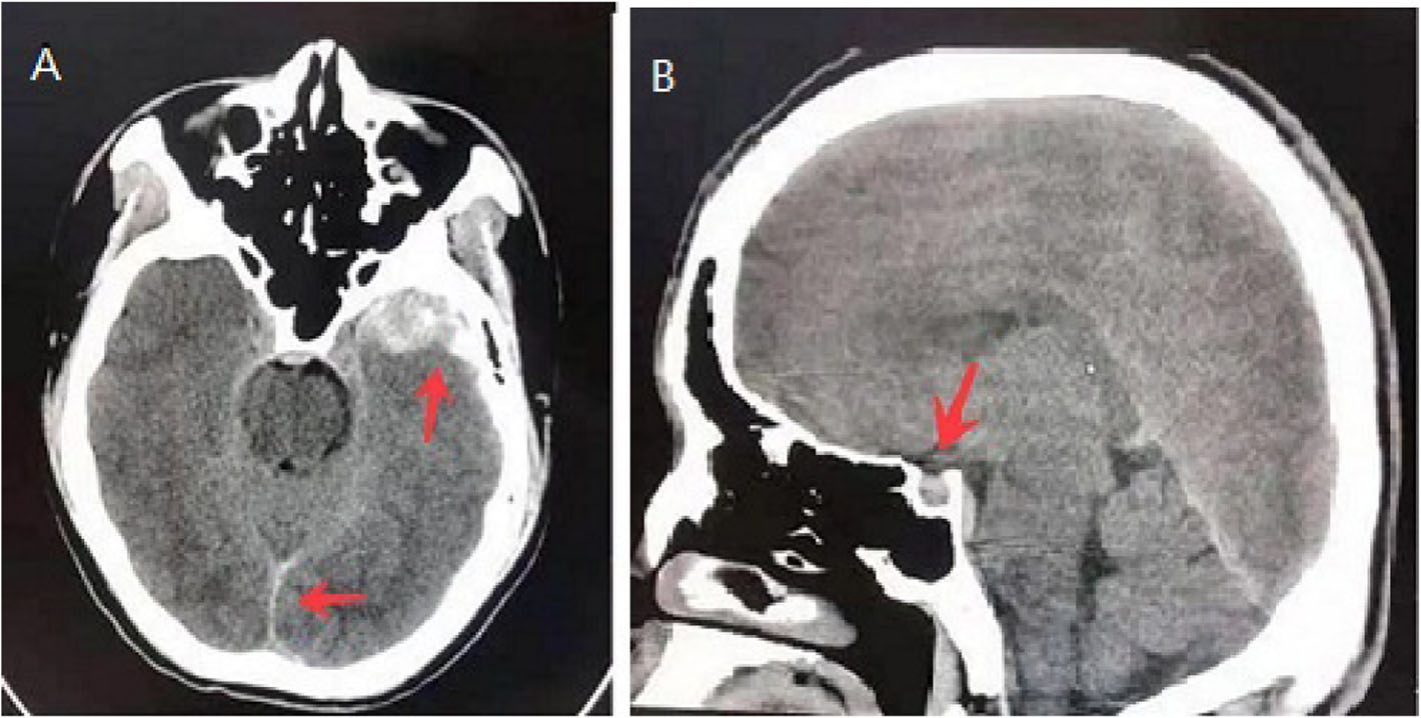
Pituitary CT at trauma 6 months ago showing subarachnoid and left subdural haemorrhage (Fig. 1**a**) and pituitary microadenoma (Fig. 1**b**)

A 70-year-old female patient with a one-year history of diabetes insipidus was transferred to the Emergency Department of our hospital due to confusion and hypotension (minimum of 70/44 mmHg, maintained by dopamine). She also had fever (maximum of 39.3 °C), chills, and cough with a small amount of white phlegm that lasted for 2 days. The patient fainted 6 months ago and fell, and the head computed tomography (CT) at that time demonstrated left frontal and occipital fractures accompanied by frontotemporal lobe cerebral contusion, subarachnoid and subdural haemorrhage (Fig. 1 a), and incidental pituitary micro adenoma (Fig. 1 b). She had menopause at 48 years old.

On examination, her body temperature was 37.9 °C, heart rate was 98 bpm, respiratory rate was 23 bpm, and blood pressure was 95/67 mmHg (maintained by dopamine). Whole body physical examination showed that she had dry skin, chapped lips, pale areola, and sparse pubic and armpit hairs. On chest examination, coarse crackles were heard in both lung bases. All other physical examination results were within normal limits.

**Fig. 2:**
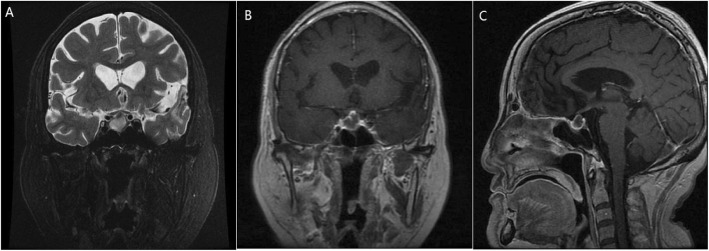
Pituitary MRI at admission showed an enlarged pituitary gland with heterogenous high signal on coronal T2 weighted image (Fig. 2**a**) and central low signal with peripheral rim enhancement on post contrast T1 weighted coronal (Fig. 2**b**) and sagittal (Fig. 2**c**) images causing mild compression on the optic chiasm, which is suggestive of acute pituitary apoplexy

Infectious work-up showed elevated neutrophil count (7.8 × 10^9^/L) and percentage (88%) as well as procalcitonin (PCT) at 0.291 ng/mL. Respiratory syncytial virus was positive, and other viruses were negative. Chest CTshowed bilateral pulmonary congestion, interstitial fibrosis, pleural effusion, and adjacent pulmonary atelectasis versus pneumonic infiltrates. Other laboratory examinations at admission revealed that the patient had yellow urine with a specific gravity of 1.015 and a volume of 4000 ml. Serum electrolyte examination showed potassium at 2.59 mmol/L, sodium at 125 mmol/L, calcium at 1.83 mmol/L, and phosphorus at 0.44 mmol/L. Laboratory adenohypophysis function examinations documented decreased thyroid, sex and adrenal hormones (Table 1). Pituitary MR imaging performed on the day of admission revealed enlarged sella measuring 15 mm × 10 mm × 9 mm with heterogenous high signal on coronal T2 weighted image (Fig. 2 a) and central low signal with peripheral rim enhancement on post contrast T1 weighted coronal (Fig. 2 b) and sagittal (Fig. 2 c) images causing mild compression on the optic chiasm, which is suggestive of acute pituitary apoplexy [8]. Thus, the patient was emergently managed for both pituitary apoplexy and pulmonary infection and was treated by hormone replacement therapy (adrenocortical hormone and levothyroxine sodium tablets), antibiotics (moxifloxacin and ganciclovir), ambroxol, and doxofylline.

**Table 1:** Laboratory tests-pituitary function

Paraclinical tests	Value	Reference value
Thyroid hormone		
Thyrotropin (TSH)	0.414 μIU/mL	0.25–5 μIU/mL
Thyroxine (T4)	2.22 μg/dL	4.2–13.5 μg/dL
Triiodothyrosine (T3)	0.550 ng/ml	0.8–2.2 ng/ml
Free-T4 (FT4)	4.98 pmol/L	9.05–25.5 pmol/L
Free-T3 (FT3)	3.19 pmol/L	2.91–9.08 pmol/L
Sex hormone		
Luteinizing hormone (LH)	< 0.1 mIU/mL	7.7–58.5 mIU/mL
Follicle-stimulating hormone (FSH)	0.927 mIU/mL	25.8–134.8 mIU/mL
Pituitary prolactin (PRL)	0.24 ng/ml	4.79–23.3 ng/ml
Oestradiol (E2)	< 18.4 pmol/L	< 18.4–201 pmol/L
Progesterone (P)	0.16 nmol/L	0.3–2.5 nmol/L
Testosterone (T)	< 0.087 nmol/L	0.101–1.42 nmol/L
Adrenal hormone		
Adrenocorticotropic hormone (ACTH)	< 1.0 pg/ml	7.2–63.3 pg/ml
COR	4.6 μg/dl	5.0–28.0 μg/dl

**Fig. 3:**
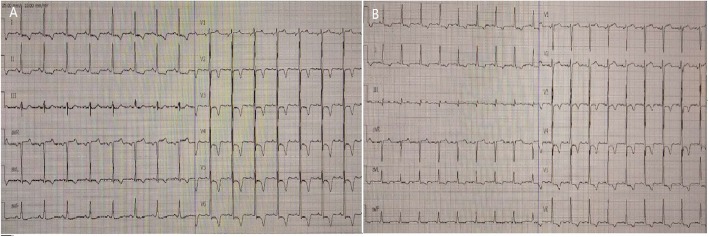
**a** ECG at admission showing T-wave inversion on the inferior and anterior wall and extended QT interval. **b** ECG at 10 days after treatment, showing that T-wave inversion partially recovered and QT interval returned to normal

**Fig. 4:**
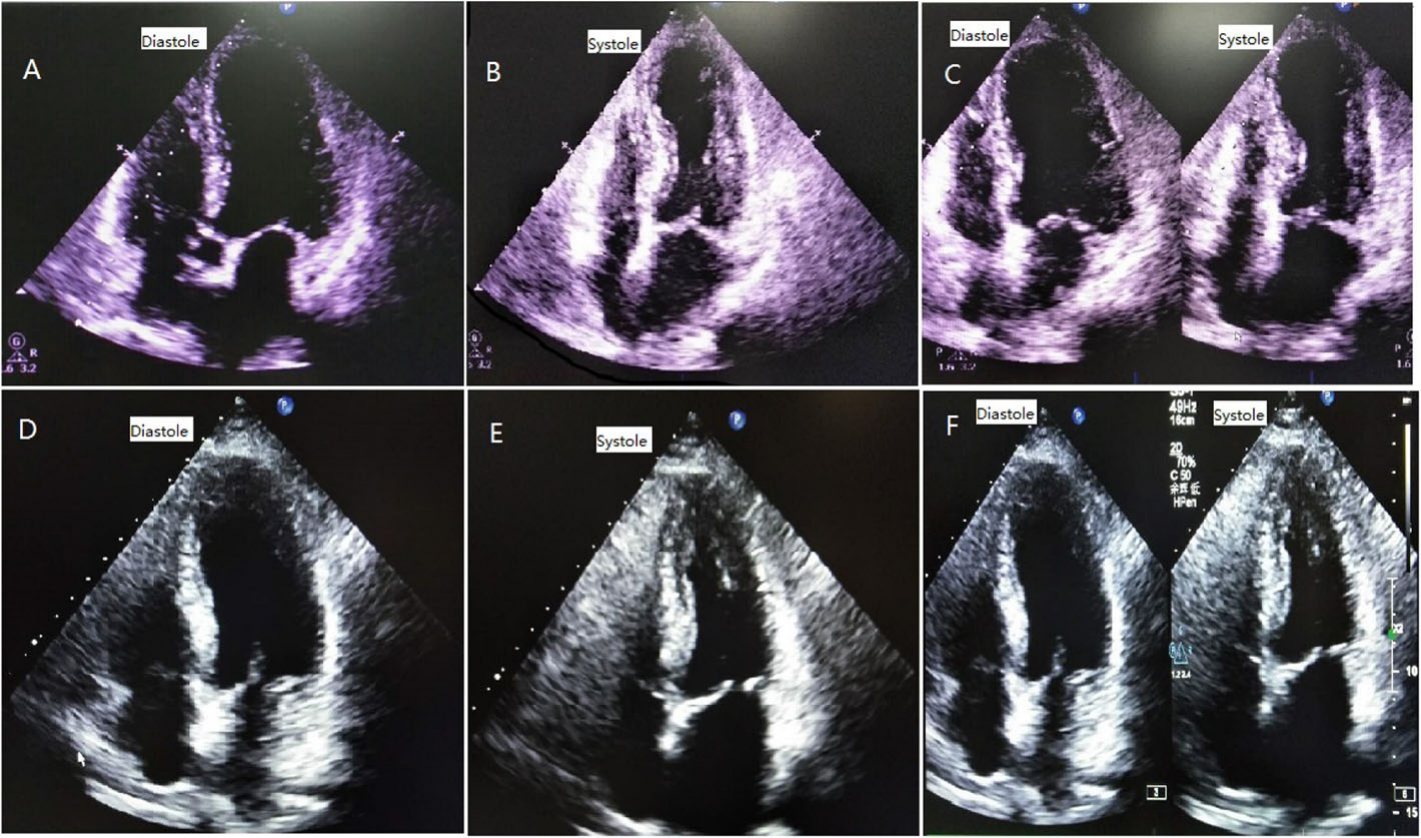
**a b c** Four-chamber view of TTE at admission showing ventricular “ballooning” caused by apical dyskinesis. **d** Four-chamber view of TTE at 7 days after treatment showing that LV apical “ballooning” was recovered. **e f** Four-chamber view of TTE two months later, showing no LV apical “ballooning” or wall motion abnormalities

**Fig. 5:**
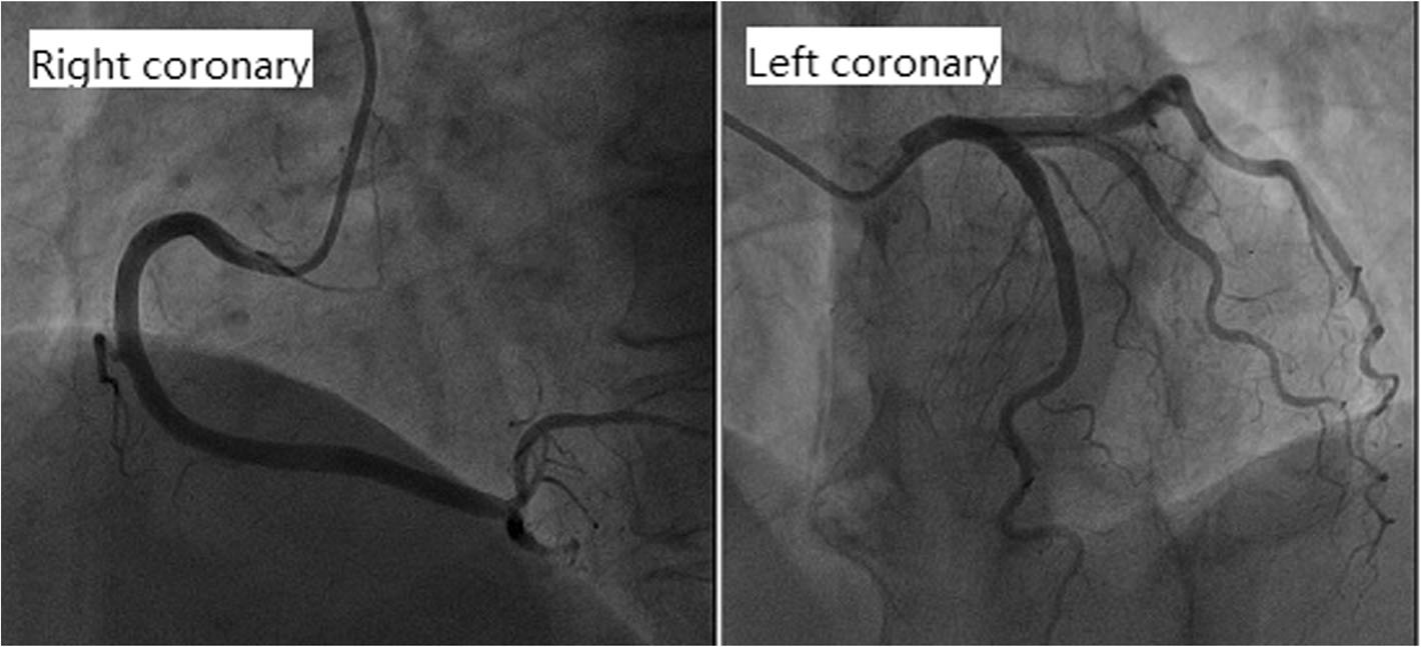
CAG demonstrating no significant coronary obstructive stenosis (both right and left coronary were nomal)

**Fig. 6:**
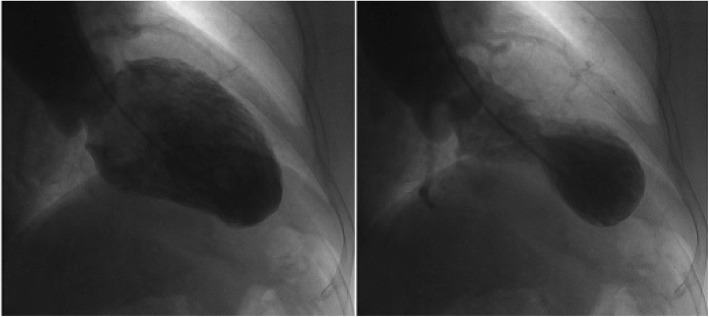
Left ventricular angiogram demonstrating that the LV had a peculiar shape (a round bottom and narrow neck), which resembles the type of bottle used in Japan for trapping octopus

Cardiac examination at admission showed elevated repeated brain natriuretic peptide precursor (Pro-BNP), C-reactive peptide (CRP), high sensitivity C-reactive peptide (hs-CRP) and troponin T (cTnT) levels (Table 2). ECG revealed T-wave inversion on the inferior and anterior walls and an extended QT interval (QT/QTc 780/762 ms) (Fig. 3 a). Emergency bedside TTE showed left ventricular ballooning, apical dyskinesia, and abnormal diastolic function (LVEF being 36% by Simpson’s method) (Fig. 4 a b c), which was considered Takotsubo cardiomyopathy. Coronary angiography (CAG) performed 2 days later found no significant coronary arterial stenosis (Fig. 5), but left ventricular angiogram demonstrated the typical apical LV wall motion abnormalities and a peculiarly shaped LV (a round bottom and narrow neck), resembling the type of bottle used in Japan for trapping octopus (Figs. 6). Therefore, the patient was diagnosed with Takotsubo cardiomyopathy and treated with angiotensin converting enzyme inhibitor (ACEI), β-blocker and L-carnitine.

**Table 2:** Laboratory tests-cardiac function

Paraclinical tests	Value	Reference value
Brain natriuretic peptide (Pro-BNP)		0–125 pg/ml
2018-01-06	3069 pg/ml	
2018-01-09	3037 pg/ml	
2018-01-12	6178 pg/ml	
2018-01-19	500.8 pg/ml	
C reactive protein (CRP)	11.1 mg/L	0–10 mg/L
Hypersensitive C reactive protein (hs-CRP)	> 10 mg/L	0–3 mg/L
Troponin T (cTnT)		0–0.014 ng/ml
2018-01-06	0.046 ng/ml	
2018-01-12	0.010 ng/ml

At 3 days after treatment, the patient’s vital signs were stable, her body temperature was 36.14°C, her heart rate was 62 bpm, her respiratory rate was 18 bpm, and her blood pressure was 124/72 mmHg.

At 10 days after treatment, Pro-BNP and cTnT levels were reduced to 500.8 pg/mL and 0.01 ng/mL, respectively (Table 1). ECG revealed that the T-wave inversion partially recovered, and the QT interval returned to normal, with QT/QTc being 440/350 ms (Fig. 3b). TTE showed recovered LV apical ballooning and normal wall motion at 10 days after treatment (Fig. 4 d).

Thirteen days after treatment, the patient suffered new abdominal pain, and her examination showed positive Murphy’s sign. Routine blood tests showed elevated neutrophil counts (8.86 × 10 [9]/L) and percentages (83%). Re-examination of Pro-BNP at the same time showed normal Pro-BNP levels. Her abdominal ultrasound was suggestive of acute cholecystitis. Thus, the patient was managed conservatively with antibiotics, and symptoms of acute cholecystitis resolved after 3 days.

At 2 months after treatment, there were no left ventricular apical ballooning or wall motion abnormalities, with LVEF at 63% by Simpson’s method (Figs. 4e and f).

## Discussion

The mechanisms of TTC are not clear but are likely related to increased plasma concentrations of catecholamines because most cases have recent excessive physical or psychological stressors [9, 10]. TTC occurs mainly in postmenopausal women [11]. Pelliccia F. et al. [12] found that 86% of TCC cases were women, and only 14% were men. These results are line with Templin C et al. [13], who found that 79.1% of TCC occurs in women over 50 years of age.

Previous studies evaluating the risk factors for TTC have suggested that the syndrome is mainly associated with a stressful event, surgery or acute clinical illness such as COPD, migraine, affective disorders, cancer, neurological disease, and psychiatric disorders but rarely with cardiovascular risk factors [13–15]. Mario S et al. [16] reported a case in which hyponatraemia can trigger Takotsubo cardiomyopathy. Either emotional or physical stress can trigger TCC development. Studies [17] have revealed that 56–89% of TTC patients suffer from stressful events (25–47% from emotional stress and 23–51% from physical stress), and 11–44% of TTC patients do not suffer from stressful events. A recent meta-analysis [12] of 1109 TTC patients showed that 39% patients had emotional stressors, 35% patients had physical stressors, and 17% patients had no stressors. In addition, the study also showed that common comorbidities of TTC patients were psychiatric disorders (24%), pulmonary diseases (15%), malignancies (10%), neurological disorders (7%), chronic kidney disease (7%), and thyroid diseases (6%). A systematic review [18] identified that bacterial sepsis was the most frequent cause of TTC. A number of TTC studies [13, 18, 19] found that 3–23% of cases were associated with surgical operation and perioperative conditions. Manfredini R et al. [20] conducted a meta-analysis on the relationship between respiratory diseases and TTC and showed that chronic obstructive pulmonary disease (COPD), acute asthmatic attack and pulmonary embolism may trigger TTC.

In this study, we report the first case of Takotsubo cardiomyopathy with hypopituitarism in the setting of pituitary apoplexy and provide evidence to support that pituitary apoplexy may be associated with myocardial stunning. The old woman suffered diabetes insipidus that may be related to prior head trauma, and cranial CT at that time revealed subarachnoid and subdural haemorrhage and pituitary micro adenoma. However, her admission investigations showed that she had decreased sex hormones, thyroid hormones and ACTH levels, hypouria, increased urine volume, and reduced electrolyte levels, suggesting that both anterior hypophysis and neurohypophysis functions were affected. The patient had suffered significant stressor such as fever and pulmonary infection before the onset of the symptoms and had emergency signs and symptoms such as hypotension and unconsciousness at the presentation, all of which conform to acute pituitary apoplexy.

The patient’s CAG showed no obvious abnormality, but the left ventricular angiogram revealed typical apical ballooning and wall motion abnormalities. TTE at admission showed LV apical ballooning, abnormal systolic function, and LVEF of 36% by Simpson’s method. In addition, the 70-year-old patient had elevated Pro-BNP, inverted T-wave and extended QT interval, all of which met the diagnostic criteria of TTC. Thus, we concluded that TTC was induced by pituitary crisis. After 10 days of treatment, the level of Pro-BNP was reduced, and T-wave inversion and QT interval extension disappeared in the ECG. In particular, there were no LV apical ballooning or left ventricular wall motion abnormalities, which suggested the recovery of the affected myocardium and explicit TTC diagnosis.

Re-examination of Pro-BNP during the attack of acute cholecystitis was in the normal range, which further confirmed that TTC was triggered by hypopituitary crisis rather than infection.

Recently, there has been increasing evidence to suggest the occurrence of TTC in patients with oestrogen deficiency, thyroid dysfunction or adrenal insufficiency [10]. The patient we describe suffered from TTC accompanied by acute pituitary apoplexy on the basis of both anterior hypophysis and neurohypophysis dysfunction. However, the mechanism remains unclear.

## Conclusions

Takotsubo cardiomyopathy is an increasingly recognized condition. Here, we report the first case of Takotsubo cardiomyopathy with pituitary apoplexy in a postmenopausal woman, significantly supporting the recognition that physical stress can trigger TTC.

## Data Availability

All the data supporting our findings are contained within the manuscript.

## Abbreviations

TTC: Takotsubo cardiomyopathy
ECG: Electrocardiogram
TTE: Transthoracic echocardiogram
LVEF: Left ventricular ejection fraction
PCT: Procalcitonin
CAG: Coronary angiography
ACEI: Angiotensin converting enzyme inhibitor
